# Development and epigenetic regulation of Atypical teratoid/rhabdoid tumors in the context of cell-of-origin and halted cell differentiation

**DOI:** 10.1093/noajnl/vdae162

**Published:** 2024-09-23

**Authors:** Laura Huhtala, Goktug Karabiyik, Kirsi J Rautajoki

**Affiliations:** Prostate Cancer Research Center, Faculty of Medicine and Health Technology, Tampere University and Tays Cancer Center, Tampere University Hospital, Tampere, Finland; Prostate Cancer Research Center, Faculty of Medicine and Health Technology, Tampere University and Tays Cancer Center, Tampere University Hospital, Tampere, Finland; Prostate Cancer Research Center, Faculty of Medicine and Health Technology, Tampere University and Tays Cancer Center, Tampere University Hospital, Tampere, Finland

**Keywords:** cell differentiation, cell-of-origin, epigenetic regulation, INI1, pediatric tumor

## Abstract

Atypical teratoid/rhabdoid tumors (AT/RTs) are aggressive brain tumors primarily observed in infants. The only characteristic, recurrent genetic aberration of AT/RTs is biallelic inactivation of SMARCB1 (or SMARCA4). These genes are members of the mSWI/SNF chromatin-remodeling complex, which regulates various developmental processes, including neural differentiation. This review explores AT/RT subgroups regarding their distinct SMARCB1 loss-of-function mechanisms, molecular features, and patient characteristics. Additionally, it addresses the ongoing debate about the oncogenic relevance of cell-of-origin, examining the influence of developmental stage and lineage commitment of the seeding cell on tumor malignancy and other characteristics. Epigenetic dysregulation, particularly through the regulation of histone modifications and DNA hypermethylation, has been shown to play an integral role in AT/RTs’ malignancy and differentiation blockage, maintaining cells in a poorly differentiated state via the insufficient activation of differentiation-related genes. Here, the differentiation blockage and its contribution to malignancy are also explored in a cellular context. Understanding these mechanisms and AT/RT heterogeneity is crucial for therapeutic improvements against AT/RTs.

**Key Points**

- Characteristics of cell-of-origin together with halted cell differentiation contribute to AT/RT malignancy.
- SWI/SNF complex has dynamic roles in neural differentiation.
- Aberrant epigenetic regulation lays the ground for AT/RT and subgroup-specific vulnerabilities.

Central nervous system (CNS) tumors originate in the brain or spinal cord and are classified into diverse cancer types according to their histological, immunohistochemical, and molecular signatures.^1^ While the incidence of most CNS tumors is higher in older individuals, certain CNS tumors, such as medulloblastoma, atypical teratoid rhabdoid tumor (AT/RT), and neuroblastoma, have a higher incidence in pediatric populations.^2^ Collectively, pediatric CNS tumors are the leading cause of cancer-related deaths in children.^3–6^ The average 5-year survival rate for pediatric malignant brain tumors in Europe is 57%.^4,7,8^ An increase in the incidence of pediatric CNS tumors has been observed in recent decades, which can be partly attributed to improvements in healthcare systems and diagnosis methods.^9,10^

AT/RTs are highly malignant and the most common intracranial neoplasms in infants, accounting for 40%–50% of CNS tumors during the first year of life.^11^ Although surgery and chemotherapy are currently utilized as therapeutic strategies, no successful therapy for AT/RT exists, resulting in poor prognosis and high recurrence rates (average overall survival is 12–20 months).^12–16^ Treatment protocols for other CNS malignancies affecting comparable age groups have been found ineffective for AT/RTs, further emphasizing the urgent need for improved therapies for patients with AT/RT.^12,17^

In this review article, we investigated AT/RTs and their molecular subgroups in terms of key transcriptional and epigenetic signatures, patient characteristics, and mechanisms driving the switch/sucrose nonfermentable (SWI/SNF)-related, matrix-associated, actin-dependent regulator of chromatin family B (SMARCB1, alias INI1) loss-of-function mutations. We further reviewed recent research on the distinct developmental stages during which AT/RT subgroups may arise, shedding light on how the cell-of-origin can contribute to AT/RT malignancy and heterogeneity. Finally, we discussed the pivotal role of differentiation blockage in AT/RT development and how it is influenced by aberrant epigenetic regulation, including both histone regulation and DNA methylation, highlighting its potential as a promising target for future therapeutic approaches.

## Aberrant Epigenetic Regulation and Neural Development in the Tumorigenesis of Embryonal Brain Tumors

Defective neural development plays a significant role in the tumorigenesis of pediatric brain tumors.^18^ Identification of the aberrant molecular mechanisms causing abnormal neural development and differentiation in these tumors is essential for identifying key players and targeting them with effective therapeutic strategies. Brain development and neural-cell differentiation are highly complex processes that produce diverse cell types in the human CNS. During embryonic development, the neural tube and the various neural stem/progenitor cells give rise to the different structures and cell types of the CNS through a series of processes including neural induction, migration, and differentiation.^19–21^ These differentiation and lineage specification events are tightly regulated by several epigenetic components and signaling pathways such as FGF, Wnt, BMP, and Notch.^22^

Although pediatric cancers exhibit a lower tumor mutational burden than that of adult cancers, numerous pediatric cancers display genetic alterations or aberrant expression patterns of epigenetic modifiers involved in development and differentiation.^8,23^ Such mutations can alter normal developmental trajectories, thereby blocking differentiation.^24^ A notable example commonly observed in AT/RTs is a loss-of-function mutation in the *SMARCB1* gene, which is a component of the mammalian SWI/SNF (mSWI/SNF) chromatin-remodeling complex.^25,26^

**Figure 1.**
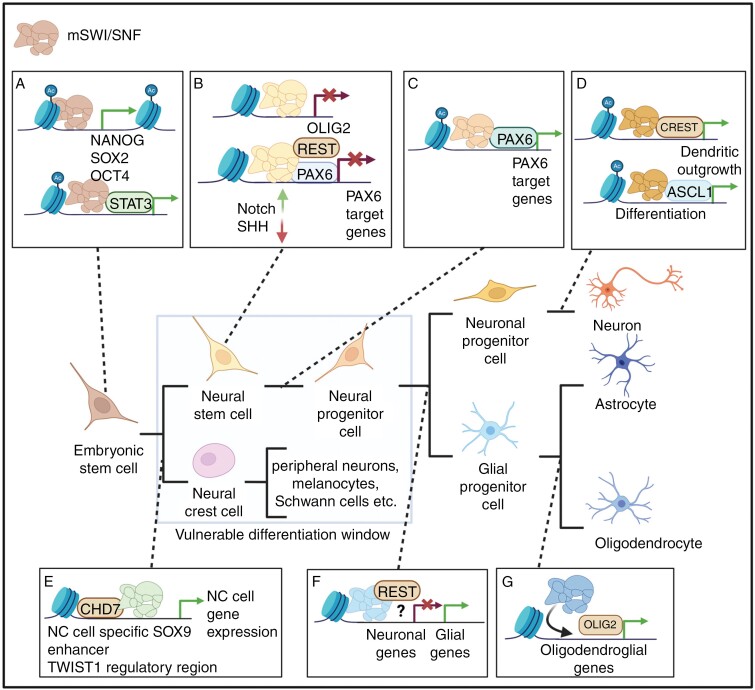
mSWI/SNF has a multitude of regulatory functions during neural cell differentiation, related to the dynamic switches in its subcomposition. Different subcompositions of mSWI/SNF are indicated with various colors. Colors are not indicative of classification into canonical, non-canonical, and pBAF. (A) During the early stages of embryonic development, mSWI/SNF is essential for pluripotency maintenance and self-renewal. mSWI/SNF increases the expression of key pluripotency genes *NANOG*, *SOX2*, and *OCT4* and stabilizes STAT3 binding to its target regions.^30^ (B) In neural stem cells, mSWI/SNF functions to maintain self-renewal and suppresses premature cell differentiation. A certain subcomposition of mSWI/SNF interacts with REST (an important repressor of neuronal differentiation) and enhances its binding to PAX6 target sites, leading to their suppression. During this developmental phase, mSWI/SNF suppresses the SHH-pathway and enhances Notch signaling, which further inhibits neuronal differentiation. Moreover, mSWI/SNF represses the expression of *OLIG2* to prevent premature oligodendrocyte development.^31–35^ (C) The switch in mSWI/SNF subcomposition acts as a trigger for neuronal differentiation. During the developmental process, the expression of different mSWI/SNF subunits undergoes changes. It has been proposed that the interaction between REST and mSWI/SNF is lost, while the interaction between mSWI/SNF and PAX6 persists, promoting the expression of PAX6 targets.^32,33,36^ (D) mSWI/SNF promotes neuronal maturation. mSWI/SNF subcomposition, specific to post-mitotic neurons, interacts with ASCL1 (an important driver of neuronal differentiation). This interaction is seemingly independent of the ASCL1 pioneer factor function. In addition, neuronal mSWI/SNF is required for CREST to bind to its target sites and promote dendritic outgrowth.^37–39^ (E) mSWI/SNF may function to promote neural crest (NC) lineage commitment and migration. In NC cells, mSWI/SNF, in collaboration with CHD7, activates NC cell-specific genes to promote cell type-specific expression.^40^ (F) mSWI/SNF may function to regulate gliogenesis. Loss of mSWI/SNF interaction with PAX6 or the loss of specific subunits leads to premature gliogenesis. Furthermore, the interaction of mSWI/SNF with REST seems vital for inducing gliogenesis, but the exact mechanisms remain unclear.^28,34,36,41^ (G) mSWI/SNF may function to promote oligodendrocyte lineage commitment. OLIG2 may target mSWI/SNF to specific genomic regions to initiate oligodendrocyte differentiation.^42^ Created in BioRender. Huhtala, L. (2024) BioRender.com/d75m420.

The mSWI/SNF complex (also known as the BAF complex) is an ATP-dependent chromatin-remodeling complex consisting of several subunits, which vary depending on whether the complex is in canonical (cBAF), non-canonical (ncBAF), or polybromo-associated (pBAF) form. This review primarily focuses on the canonical form, and “mSWI/SNF” is used to refer to the canonical form hereafter unless mentioned otherwise. mSWI/SNF plays important roles in neurogenesis, neural migration, and maturation by regulating the transcriptional accessibility of a large number of genes (Figure 1).^27,28^ While discovered in yeast, the mSWI/SNF complex has been conserved throughout evolution and identified in a wide range of eukaryotic organisms.^29^

The mSWI/SNF complex operates by repositioning and restructuring nucleosomes, thereby remodeling the chromatin structure to allow enhanced access to nucleosomal DNA. Through its interaction with histone acetyltransferases such as CREBBP/p300, mSWI/SNF facilitates the acetylation of H3K27. This leads to increased gene and enhancer activity and antagonizes the effect of the polycomb repressive complex (PRC2), which is responsible for the trimethylation of H3K27.^43–45^ The mSWI/SNF complex exhibits a strikingly dynamic composition throughout neural differentiation (Figure 1). While some core subunits appear to be constitutive parts of the complex, the expression of other subunits is under tight temporal regulation. Switches in mSWI/SNF subunit composition play a significant role in the progression of neural differentiation and lineage commitment (Figure 1).^28,46^

**Figure 2.**
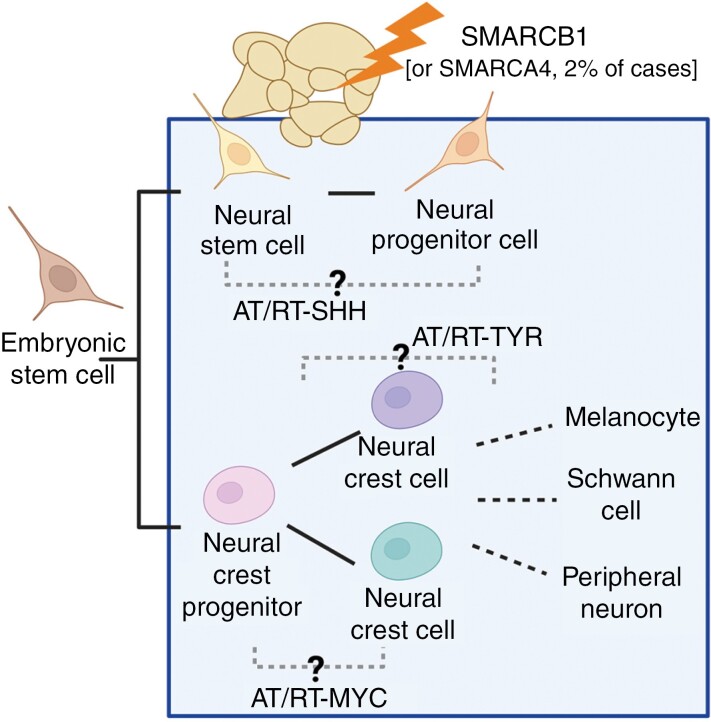
SMARCB1 inactivation leads to the development of different AT/RT subgroups depending on the phase of cell development. The neural cell differentiation window during which SMARCB1 inactivation leads to AT/RT development spans from neural stem cells to neural progenitor cells. During other stages of differentiation, SMARCB1 loss is lethal (before the neural stem cell phase) or does not similarly affect cell differentiation (more mature cells). The cell of origin for SMARCA4 inactivated tumors is still unclear due to the shortage of data. Nonetheless, different AT/RT subgroups are likely to arise from different developmental phases, which contributes to their epigenetic and transcriptomic characteristics, but more research is needed to conclusively determine the cells of origin in each subgroup. Created in BioRender. Huhtala, L. (2024) BioRender.com/u10p943.

Dysfunctional mSWI/SNF complex has been reported in around 20% of all cancer cases.^29,47^ Interestingly, mutations of the mSWI/SNF complex subunits observed in neurodevelopmental disorders are usually missense mutations, while mutations resulting in the deletion or silencing of the mSWI/SNF complex subunits are associated with cancer, including AT/RTs (Figure 2).^48^ Despite the fact that SMARCA4 and SMARCA2 are responsible for the catalytic activity of mSWI/SNF, mutations leading to carcinogenesis are frequently observed in the non-catalytic subunits of the complex, such as ARID1A, ARID1B, or SMARCB1.

## AT/RT Subgroups Show Distinct Features

**Figure 3.**
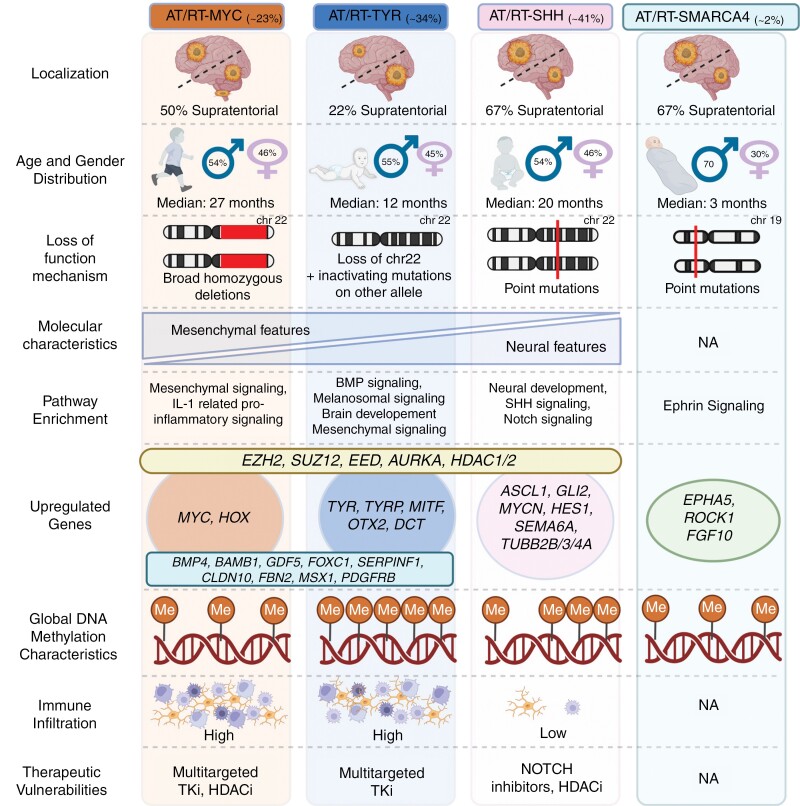
Molecular and clinical characteristics of AT/RT subgroups. AT/RTs are classified into 4 distinct subgroups: AT/RT-MYC, AT/RT-TYR, AT/RT-SHH, and AT/RT-SMARCA4, the 3 first of which are more extensively characterized. These subgroups exhibit diverse characteristics in terms of location, age, and sex distribution. Loss of SMARCB1 function, as a hallmark of AT/RTs, differs between subgroups (while loss of SMARCA4 is observed on AT/RT-SMARCA4 instead). Furthermore, each subgroup is associated with distinct pathway enrichments, although some genes, such as *EZH2*, *SUZ12*, *EED*, *AURKA*, and *HDAC1/2*, are commonly upregulated across major AT/RT subgroups. Immune infiltration levels vary among subgroups, with AT/RT-SHH exhibiting the lowest levels of immune infiltration. Furthermore, recent studies have identified subgroup-specific therapeutic vulnerabilities, providing potential targets for new treatment strategies. Created in BioRender. Rautajoki, K. (2024) BioRender.com/t07a339.

Biallelic inactivation of *SMARCB1* is the only prevalent genetic aberration observed in AT/RTs (in rare cases, *SMARCB1* is retained, whereas *SMARCA4* function is lost^49^). Despite the low genetic variation, emerging research has provided insights into the transcriptomic and epigenetic heterogeneity of AT/RTs, upon which the field has reached a consensus on 3 distinct molecular subgroups: AT/RT-TYR, AT/RT-SHH, and AT/RT-MYC (Figure 3).^50–54^ More recently, SMARCA4 deficient AT/RTs (AT/RT-SMARCA4s), which consist of 2% of AT/RT cases, were suggested as a distinct molecular subgroup (Figure 3).^55,56^ The 3 major subgroups share little similarities; only a few genes (*EZH2*, *SUZ12*, *EED*, *AURKA*, *HDAC1/2*) have been shown to be overexpressed in these subgroups compared to normal brain tissue.^52,53^ AT/RT-SHH exhibits enrichment of genes related to axonal guidance (*SEMA6A*, *TUBB2B/3/4A*) and neural development (*FABP7*, *LHX2*, *MEIS2*) together with overexpression of SHH (*MYCN*, *GLI2*, *PTCH1*, *BOC*) and Notch (*ASCL1*, *HES5/6*, *DLL1/3*, *DTX1*) signaling pathway genes.^50–53^ Gene enrichment analyses have revealed only a minor overlap between AT/RT-SHH and the other 2 major subgroups. While AT/RT-MYC and AT/RT-TYR show differences in differentially regulated genes, tumor location, and median patient age, they display some common features as well.^51,53^ Notably, pathways associated with the immune response and genes related to BMP signaling (*BMP4*, *BAMB1*, *GDF5*, *FOXC1*) and mesenchymal differentiation (*SERPINF1*, *CLDN10*, *FBN2*, *MSX1*, *PDGFRB*) are enriched in both AT/RT-MYC and AT/RT-TYR.^51,53,57^ Overexpression of tyrosinase (*TYR*), which is not expressed in AT/RT-SHH or AT/RT-MYC, has been established as the most distinguishable marker of the AT/RT-TYR subgroup. Moreover, the expression of melanosomal genes (*MITF*, *DCT*, *TYRP*) and the brain development-related gene *OTX2* are associated with this subgroup. Similarly, higher expression of *MYC* and genes in the HOX cluster distinguishes AT/RT-MYC from the other major subgroups.^51–53,57^ Highlighting the importance of subgroup-defining pathway activation, emerging research has hinted at subgroup-specific vulnerabilities, such as tyrosine kinase inhibitors for AT/RT-MYC and Notch inhibitors for AT/RT-SHH, suggesting potential for subgroup-specific targeted therapies in the future.^51,58,59^ SMARCA4 deficient AT/RTs were mostly reported within the AT/RT-SHH subgroup according to previously utilized tumor classifiers.^56^ However, with the accumulation of more data, it has been shown that AT/RT-SMARCA4 differs from the other subgroups based on transcriptomic profile.^56^ AT/RT-SMARCA4 tumors exhibit distinct differentially regulated genes, most notably the upregulation of Ephrin forward signaling, which plays a crucial role in axon guidance and neuronal migration during brain development.^56,60^ Hence, it was suggested to be one of the key pathways promoting tumorigenesis in AT/RT-SMARCA4.^56^ Additionally, AT/RT-SMARCA4 displays upregulation of *EPHA5*, *ROCK1,* and *FGF10* as well as downregulation of *GLI2*, *MITF*, *MYC,* and *DMRT2*, which exhibit upregulation in some of the major AT/RT subgroups as discussed above.^56^

Distinctive gene expression profiles of the aforementioned subgroups are rooted in differences in the epigenome, as AT/RT-MYC, AT/RT-SHH, AT/RT-TYR, and AT/RT-SMARCA4 can be distinguished from each other based on DNA methylation.^51–53,56,61^ These findings suggest that DNA methylation is associated with different gene expression patterns and phenotypes. AT/RT-TYR and AT/RT-SHH exhibit global and promoter DNA hypermethylation compared to other pediatric brain tumors, as well as normal pediatric and adult cerebella (Figure 3). On the contrary, the DNA methylation levels of AT/RT-MYC resemble those of other pediatric brain tumors (medulloblastoma, glioblastoma, and ependymoma), exhibiting general hypomethylation compared to the normal pediatric brain (Figure 3).^52,57^ Moreover, the DNA methylation profile of AT/RT-MYC resembles that of extracranial malignant rhabdoid tumors (ecMRT).^53,57^ Nonetheless, while AT/RT-MYC exhibits less global DNA methylation than the other major subgroups, previous research has identified specific regions that are consistently methylated in all of the major subgroups, indicating shared features.^62^ While AT/RT-SMARCA4 also exhibits globally hypomethylated DNA, distinguishing it from the hypermethylated SHH and TYR subgroups, it displays differences to AT/RT-MYC and ecMRTs. However, due to limited research, consequent from the rarity of the phenotype, the features potentially shared across all subgroups require further investigation.^56^

In addition to the aforementioned subgroup-identifying features, the SMARCB1 deficient subgroups exhibit differences in the mechanism underlying the loss of *SMARCB1*. The prevalent mechanism for SMARCB1 inactivation in AT/RT-TYR is the partial or complete loss of one copy of chromosome 22 with co-occurring or preexisting inactivation of the other allele.^51–53^ Copy number alterations in chr22 and deletions of *SMARCB1* are less frequently observed in AT/RT-SHH, where heterozygous point mutations appear to be the common cause of SMARCB1 loss-of-function.^51–53,57^ In contrast, point mutations have been rarely observed in AT/RT-MYC subgroup, in which broad homozygous deletions underlie the silencing of *SMARCB1*.^51–53,57^ In approximately 30% of patients, *SMARCB1* mutation already existing in the germline, a condition known as rhabdoid tumor predisposition syndrome 1.^17,55,63,64^ SMARCA4 deficient tumors, however, are associated with higher frequency of inherited germline mutations, and thus with a rhabdoid tumor predisposition syndrome 2.^17,63,64^ Furthermore, the AT/RT subgroups, including AT/RT-SMARCA4, are associated with distinct tumor locations and patient age (Figure 3).^17,51–53,55,56,65,66^ AT/RT-SHH has recently been further divided into 3 subtypes, 1A, 1B, and 2, which differ with respect to location and patient age.^53,65,66^ Studies have also indicated higher immune cell infiltration in AT/RT-MYC and AT/RT-TYR than in AT/RT-SHH (Figure 3).^57,67,68^

The association between AT/RT subgroups and survival has been inconclusive thus far, as some studies have found no significant association^66,67,69^ while others have indicated a more positive prognosis for the AT/RT-TYR subgroup.^54^ Furthermore, AT/RT-SMARCA4 patients have been reported to exhibit worse prognosis than those in other subgroups.^55^ Some of the differences between the SMARCB1 deficient subgroups might be related to variations in metastatic behavior; AT/RT-SHH, which is most prone to metastasis, also exhibits worse outcomes. However, when comparing infants with no metastatic disease, none of the major subgroups were significantly associated with patient outcomes.^69^

While AT/RT-SMARCA4 consists of an important subgroup of AT/RT, due to the rarity of this tumor phenotype and the consequent small number of analyzed samples and restricted data, this review will focus on the 3 major subgroups, consisting of the majority of AT/RTs, from this point onward.

## Cell-of-Origin Contributes to AT/RT Heterogeneity and Malignancy

There is an active scientific discussion on whether the cell-of-origin or accumulating genetic alterations better explain the tumor phenotype. Moreover, there are varying opinions on whether the cell-of-origin actively contributes to the tumor phenotype and malignancy. AT/RTs provide a unique setting to discuss the functional significance of the cell-of-origin, as their mutational burden is almost identical across the board, yet the aforementioned subgroup-specific characteristics are observed. The cell-of-origin, as an explanatory factor for distinct subgroups, has been the subject of intensive investigation. Recent studies on organoid and mouse models have elucidated a specific early developmental time frame (mouse embryonic days 6 to 10) during which *SMARCB1* inactivation leads to the formation of malignant rhabdoid tumors, thus narrowing the pool of potential cells of origin to early development (Figure 2).^58,70–72^

Single-cell and bulk transcriptome analyses of AT/RT-MYC have strongly hinted at non-neuroectodermal, more specifically neural crest (NC), origins of these tumors (Figure 2).^58,72–74^ However, a recent mouse model study suggested that fetal primordial germ cells (PGCs), a population of multipotent and epigenetically highly dynamic cells that normally give rise to gametes,^75^ could be the origin of AT/RT-MYCs.^70^ The notion was based on similarities in gene expression profiles and the potential of *SMARCB1* KO PGCs to give rise to intra and extracranial MRTs.^70^ However, NC cells and PGCs belong to completely different developmental lineages, with PGCs having no relation to ectodermal development.^75^ Furthermore, PGCs exhibit highly active MYC signaling,^76^ which could explain the observed similarities. Thus, consensus on the origin of AT/RT-MYC remains elusive, although NC lineage has received more support.^58,72–74^ The evidence seems to point towards more migratory cell types in early development, which would be reasonable given the extracranial location of MYC-like MRTs and the spinal location of some AT/RT-MYC tumors.^57^ The development of AT/RT-MYC versus ecMRT could be a matter of timing, where a mutation in the NC specification phase (marked by strong expression of MYC) would lead to brain-resident AT/RT-MYC, whereas a mutation occurring after the delamination phase in already migratory NC cells would result in ecMRT.^21^

As AT/RT-SHH is restricted to the brain, it would be natural for early neuronal lineage progenitor cells to be the possible origin for this subgroup. Indeed, multiple pathway enrichment analyses have indicated a more neurogenic signature for AT/RT-SHH, in which brain development and other various neuronal differentiation-related processes are more active than in the other subgroups (Figure 2, Figure 3).^51–53,57,58,65,70,72^ Neuronal progenitors, more specifically progenitor cells from the ganglionic eminence or midbrain-hindbrain boundary, have been suggested as ancestors of 2 different AT/RT-SHH subtypes based on gene expression studies involving human tumors and murine AT/RT models (Figure 2).^58^ Cells from the ganglionic eminence may give rise to SHH tumors located in the basal ganglia and intraventricular regions, whereas midbrain-hindbrain progenitors were proposed as the origin of SHH tumors in the cerebellar anterior lobe. Lobón-Iglesias et al. also suggested that a mechanism hijacking normal neuronal differentiation would underlie type 2 AT/RT-SHH development from the midbrain-hindbrain progenitors. They showed that *SMARCB1* loss in neural progenitor cells leads to a loss of expression of neuronal differentiation markers and increased expression of the neuronal repressor REST, stemness markers, and Notch receptors. Consequently, the surrounding normal (*SMARCB1* retained) progenitor cells expressing Notch ligands trigger the activation of the Notch pathway in mutated cells, thus inhibiting differentiation. Moreover, In-vitro modeling of Notch inhibition in AT/RT-SHH cells showed reduced expression of stemness markers with simultaneous upregulation of the neuronal progenitor markers *NEUROG2* and *NEUROD1*.^58^

It is intriguing to speculate whether the cell-of-origin or the type of *SMARCB1* mutation is responsible for subgroup-related differences. Cribriform neuroepithelial tumors (CRINETs), rare non-rhabdoid tumors with *SMARCB1* loss accompanied by high Tyrosinase expression, strikingly resemble AT/RT-TYR based on the DNA methylation profile and gene expression.^77^ Moreover, the tumors share another feature: both harbor large heterozygous losses of chr22, with accompanying mutations in *SMARCB1* in the other allele, which is uncommon in other AT/RT subgroups.^77^ Interestingly, CRINETs have a significantly better prognosis than AT/RT-TYR.^77^ Since molecular differences between CRINETs and AT/RT-TYR are sparse, more studies on these tumor types are crucial to elucidate the underlying mechanisms responsible for the differences in prognosis. While the cell-of-origin for these tumor types remains unresolved, it is intriguing that a very similar mutation event in the *SMARCB1* locus is able to promote tumor types with differing prognoses. Furthermore, the existence of a restricted developmental window for AT/RT development suggests the importance of the cell of origin. If the same inactivation has no effect on differentiated cells, but in progenitor cells, it leads to malignancy, it is undeniable that the cell-of-origin contributes to the tumor phenotype.

The fact that the AT/RT subgroups are associated with different *SMARCB1* alterations suggests that the mutation event itself plays a relevant role in the tumor cell phenotype. However, *SMARCB1* inactivation has driven the development of both MYC and SHH subgroup tumors in mouse models, despite the same mechanism of inactivation.^58,70,72^ Interestingly, AT/RT-SHH was shown to exhibit a more restricted developmental window, as tumors developed exclusively via *SMARCB1* inactivation during mouse embryonic days 6 and 7, whereas AT/RT-MYC was achieved with *SMARCB1* inactivation on days 6 to 10.^58^ Of note, *SMARCB1* inactivation in induced pluripotent stem cells (iPSCs) and during mouse embryonic days 1 to 5 has been shown to be lethal.^71,72^ Furthermore, a mouse model study demonstrated the occurrence of various distinct phenotypes when *SMARCB1* inactivation was linked to key regulators that exhibit time- and cell-type-specific expression during neural development.^70^ These findings highlight the fine-tuned complexity of the process and indicate that AT/RT development is dependent on several factors and not solely on the *SMARCB1* inactivation mechanism, in line with the complex functions of the mSWI/SNF during neural differentiation (Figure 1). Thus, AT/RT development can be considered a “3-hit model”, where the differentiation stage, cell phenotype, and a single mutation event collectively need to align to induce tumor development. While AT/RT is quite a unique tumor type with a single inactivation event underlying cancer development, some similar low mutational-burden tumors exist among other pediatric brain cancers. The differentiation stage, cell-of-origin, and mutation status play significant roles in the tumorigenesis of these tumors, as well, making the 3-hit model applicable to also other developmental tumors with low mutational burden.^78^ First, as demonstrated in various studies, the mutation must occur in the progenitor cells. Second, it is possible that a specific mutation event in a particular progenitor cell is required. For instance, a mutation event common to AT/RT-MYC would need to occur in the NC for the tumor to develop, whereas a mutation in any other cell type would lead to a non-tumor or lethal phenotype. Similarly, a mutation event common to AT/RT-SHH would need to occur in the neural progenitor cells to produce malignant cells. However, the ongoing research does not rule out the possibility that the type of *SMARCB1* alteration would contribute to subgroup distinction. It can be hypothesized that different alterations in chr22 cause slight nuances in the epigenome and gene regulation of malignant cells that drive the distinct subgroup features. Interestingly, no functional in vivo or in vitro models of AT/RT-TYR, characterized by the hemizygous loss of chr22, have been achieved.^53,58,72^ While the less aggressive behavior of AT/RT-TYR tumors might contribute to that, the reason why the experimental setups only give rise to AT/RT-MYC and AT/RT-SHH is intriguing. The expression of melanosomal genes in AT/RT-TYR might suggest that the cell-of-origin could be a NC cell committed towards the melanosomal lineage (Figure 2); hence, this would explain why in vitro models restricted to studying neural differentiation would be unable to give rise to AT/RT-TYR subgroup tumors. However, as in vivo models also fail to produce the TYR subgroup, the mechanisms underlying the development of these tumors remain unresolved.

Although the exact relative significance of the mutation event and the cell-of-origin is to be uncovered, it is evident that AT/RTs hijack the regulation of neural progenitors or stem cells. Silencing genes related to brain development and neural differentiation, together with global DNA hypermethylation, blocks neural cell differentiation. While the relatively stable epigenome of more differentiated cells remains unaffected, the delicate temporal regulation of differentiation in progenitor cells is susceptible to distortions. Thus, in the context of AT/RT, the cell-of-origin can be considered a contributor to malignancy rather than just an ancestor. Therefore, it is important to pinpoint the exact mechanisms of the neural differentiation process that ceases to function and remains at a standstill upon *SMARCB1* loss, as well as those that become aberrant in this context.

## Aberrant Epigenetic Regulation and Its Treatment Potential in AT/RTs

As discussed, cells in the early stages of neural differentiation are affected more by *SMARCB1* loss in terms of rhabdoid tumor development than later committed neural progenitors. The enrichment of neural differentiation-related pathways and genes in all AT/RT subgroups further indicates that halted cell differentiation is at the core of AT/RT development and malignancy. Interestingly, SMARCB1 is reportedly a part of the mSWI/SNF complex throughout neural differentiation and belongs to the complex core.^28,31,46^ Therefore, the existence of a vulnerable differentiation window for AT/RT development does not result from the temporal expression window of SMARCB1. This emphasizes the critical role of specific stages of cell differentiation within a broader cellular context extending beyond the association with *SMARCB1* expression during the emergence of AT/RTs.^71,72^ Recent advancements in the understanding of AT/RTs and their epigenetic backgrounds have opened new directions for therapeutic interventions. There is a growing interest in targeting the differentiation blockage in AT/RTs^29^ similar to recent therapeutic approaches in hematological malignancies, where induced cellular differentiation leads to apoptosis.^79^ The epigenetic dysregulation observed in AT/RTs leading to blockage of differentiation is currently under investigation, and emerging insights have suggested a dual mechanism involving both DNA methylation and histone modifications, both of which are discussed below.

## Histone Regulation on AT/RT Differentiation Blockage

**Figure 4.**
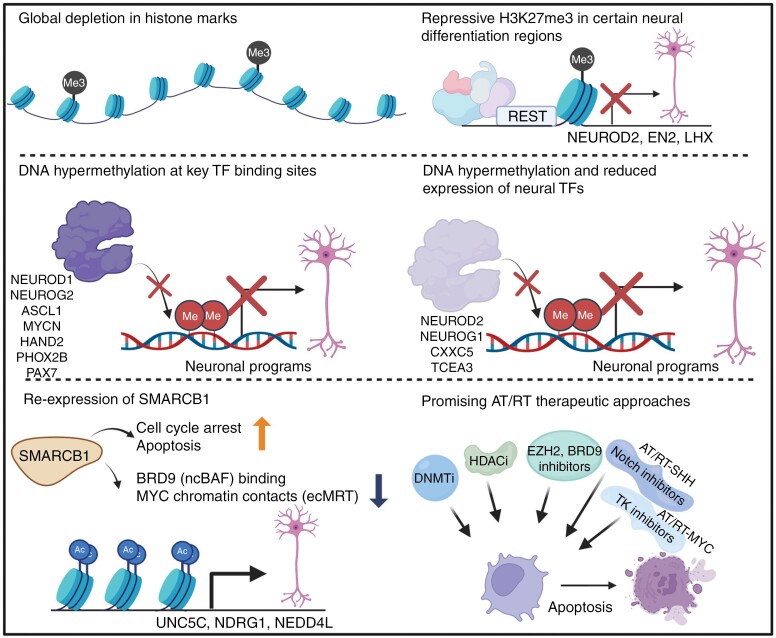
Mechanisms of suppressed neural differentiation in SMARCB1 deficient AT/RT and the key genes and transcription factors known to exhibit dysregulation. Both deregulated histone modifications and DNA hypermethylation affect the differentiation blockage observed in AT/RTs. The re-expression of SMARCB1 in AT/RT cells leads to H3K27 acetylation, detachment of the ncBAF complex from the chromatin, and cell cycle arrest. While many efforts have been made to overcome the disease, the inhibitors, including DNA methyltransferase (DNMTi) and histone deacetylase (HDACi) inhibitors as well as inhibitors against BRD9, EZH2, Notch pathway, and tyrosine kinases (TK), have shown promising and partly subgroup-specific efficacy in AT/RT. Created in BioRender. Rautajoki, K. (2024) BioRender.com/g54p437.

The mSWI/SNF complex is a crucial epigenetic regulator of several developmental processes. In AT/RT, the inactivation of *SMARCB1* leads to a global loss of the H3K27ac mark, associated with open chromatin, which is, unexpectedly, not counterbalanced by a global increase in the repressive H3K27me3 mark (Figure 4).^80^ Instead, it leads to a prevailing quiescent state in the AT/RT genome, marked by the absence of histone marks (Figure 4).^80^ However, genomic locations, where SMARCB1 occupation (and active chromatin state) is detected in normal pediatric brain tissue, only partly exhibit a switch to quiescent chromatin. These regions more commonly exhibit a PRC2-repressed chromatin state, characterized by the presence of EZH2 and repressive H3K27me3 (Figure 4). Genes suppressed via this mechanism include multiple neural differentiation-related genes such as *NEUROD2* (a pioneer factor and master regulator of neuronal differentiation and neurogenesis^81,82^), *EN2* (neurite outgrowth during development^83,84^), and *LHX1* (neural cell migration during development, differentiation of neurons with specified functions^85,86^; Figure 4). Re-expression of SMARCB1 in AT/RT cell lines induces a global increase in the active H3K27ac mark, accompanied by the upregulation of genes that participate in neural development, including UNC5C (axon guidance^87^), NDRG1 (downstream factor of MYCN, important for myelination^88^), and NEDD4L (cortex development, neural migration, PAX6 degradation^89,90^; Figure 4). In addition, G1 cell cycle arrest leading to apoptosis is observed upon SMARCB1 re-expression.^80,91^ (Figure 4). These findings suggest that some neuronal differentiation-related genes which normally exhibit SMARCB1 binding, are suppressed by PRC2/EZH2-mediated H3K27me3 deposition in AT/RTs.

Interestingly, a large group of protein-coding genes with EZH2 occupancy in their promoters do not display the repressive H3K27me3 mark in AT/RTs.^80^ SMARCA4 binding on those regions has been proposed as a marker of residual mSWI/SNF activity.^80,92^ Co-localization of the REST complex, a known inhibitor of neural cell differentiation,^93^ with EZH2, has been reported on promoters marked with H3K27me3. The presence of REST at the promoters disrupts SMARCA4 binding, thereby facilitating H3K27me3 deposition^80^ (Figure 4). Supporting the importance of residual SMARCA4 activity, *SMARCA4* knockdown on rhabdoid cell lines has been shown to induce apoptosis.^44,92^ Recent studies on ecMRTs have further elucidated the role of residual mSWI/SNF activity. Upon SMARCB1 loss, the ncBAF, a subcomposition of mSWI/SNF that does not include SMARCB1, interacts with additional chromatin regions, including the MYC-locus, facilitating open chromatin conformation and enhancer activity.^94^ ncBAF binding to these regions is dependent on the SMARCB1 loss, evident from SS18 (cBAF and ncBAF) and BRD9 (ncBAF) binding greatly diminishing upon SMARCB1 reconstitution^94^ (Figure 4). These results indicate that SMARCB1 loss facilitates changes in the chromatin landscape also by inducing an imbalance between cBAF and ncBAF and that this mechanism would be responsible for MYC upregulation on ecMRTs.^94^ Moreover, a genome-wide CRISPR-Cas9 screen study demonstrated that SMARCB1 deficient MRT cells are dependent on BRD9 expression.^95^ Interestingly, in MRT cells, BRD9 and SMARCA4 binding was shown to overlap to a large degree.^96^ The phenomena was diminished upon SMARCB1 reconstitution, and BRD9 inhibition significantly reduced SMARCA4 binding.^96^ It remains elusive whether the aforementioned^80^ residual SMARCA4 activity in AT/RT also relates to the deregulated function of ncBAF, but this is certainly an intriguing notion. A recent study on ecMRT demonstrated that inhibiting DCAF5 rescues the binding of mSWI/SNF lacking SMARCB1 to normal mSWI/SNF target loci, thus restoring the function of the complex and cell differentiation. DCAF5 functions to degrade misassembled mSWI/SNF. They found this process not to be interfering with the function of ncBAF, indicating another layer to the complex regulation and dynamics of the mSWI/SNF function.^97^

While it seems that other mSWI/SNF subunits and/or non-canonical compositions exhibit aberrant behavior after the loss of SMARCB1 in some instances, it remains unclear what are the underlying mechanisms. Furthermore, the question of whether the mechanism of the distorted function of the ncBAF differs (or even exists) between AT/RT subgroups remains unresolved. And, to further add to the complexity, it was shown that in the absence of SMARCB1, ecMRT cells become dependent on p300-CBP acetyltransferases, evident from synthetic lethality of dual inhibition of both p300 and CBP in SMARCB1 deficient, but not in SMARCB1 reconstituted ecMRT model.^98^

Recently, the antagonistic relationship between the mSWI/SNF and PRC2 complexes was used to develop novel therapeutic strategies for AT/RTs^99^ (Figure 4). *EZH2* knockdown in *SMARCB1* deficient ecMRT tumors decreased the tumor growth rate,^100^ while targeting EZH2 with Ribavirin^101^ or DZNep^102^ led to increased survival in orthotopic AT/RT xenograft models. The same effect was also observed upon inhibition of EZH2 via the small-molecule inhibitor GSK126 in combination with the BET inhibitor JQ1, which has previously been shown to be effective, especially against AT/RT-MYC.^103^ Similarly, tazemetostat, a selective EZH2 inhibitor, completed a phase I trial with promising effects in a subpopulation of patients with AT/RT.^104,105^ However, as reported by Erkek et al., the inhibition of EZH2 alone might increase the risk of further activation of genes already active in AT/RTs.^80^ Additionally, in a similar Phase II study, in which tazemetostat was tested against *SMARCB1* deficient tumors, therapeutic efficacy was observed only in a subpopulation of patients, supporting the patient-specific effects of EZH2 inhibition.^106^

Similarly, histone deacetylase (HDAC) inhibitors have emerged as potential treatment strategies for AT/RTs, as a single treatment or in combination with EZH2 inhibition, after they have previously shown successful responses in prostate cancer^107^ and neuroblastoma^108^ (Figure 4). Overall, HDAC inhibitors demonstrated an anti-proliferative effect on AT/RTs. Depsipeptide treatment induces an increase in global acetylation levels and apoptosis.^109,110^ Similarly, panobinostat, a broad-spectrum HDAC inhibitor, trigger cell differentiation and inhibits tumor growth in ecMRT models.^111^ Interestingly, while the broad-range HDAC inhibitor vorinostat did not affect tumor growth in an ecMRT xenograft model when administered in combination with irradiation, the treatment showed greater efficacy against rhabdoid tumors.^112^

Despite the contribution of aberrant PRC2 function to differentiation blockage (evident from the increase in the repressive H3K27me3 mark at SMARCB1 binding sites in AT/RT compared to the normal pediatric brain), the global quiescent state of the AT/RT genome and its influence cannot be overlooked. Moreover, as DNA methylation appears to contribute to subgroup-defining features and phenotypes, it is valuable to consider oncogenic epigenetic mechanisms other than histone remodeling in AT/RTs.

## Role of DNA Methylation on AT/RT Differentiation Blockage

Multiple studies have demonstrated global DNA hypermethylation in AT/RTs, suggesting DNA methylation-driven suppression of neural differentiation (Figure 3, Figure 4).^52,57,62^ Recently, a more detailed perspective on AT/RT DNA methylation was revealed, possibly elucidating how the global quiescent state may play a role in deregulating differentiation-related pathways.^62^ Pekkarinen et al. compared AT/RT DNA methylation characteristics with those of 2 other pediatric cancers (choroid plexus and medulloblastoma), pluripotent stem cells, and the normal fetal brain. They demonstrated that patients with AT/RT exhibited DNA hypermethylation at the binding sites of NEUROD1, NEUROG2, and ASCL1, pioneer factors functioning as master regulators of neurogenesis and neuronal differentiation, as well as at the binding sites of MYCN (regulation of neural progenitor proliferation and differentiation, maintenance of NC cells^113,114^), HAND2 (specification of noradrenergic sympathetic ganglion neurons^115^), PHOX2B (important for autonomic NC derivatives, cooperation with ASCL1),^116,117^ and PAX7 (specification of NC^118^) (Figure 4).^37,62,81,119^ Furthermore, DNA hypermethylation in the regulatory regions with a simultaneously suppressed expression was observed for NEUROG1 and NEUROD2, both crucial pioneer factors regulating neurogenesis and neuronal differentiation,^81,119^ as well as for CXXC5 (regulation of WNT pathway, regulation of myelination and differentiation of neural stem cells into a variety of cellular fates^120,121^), and TCEA3 (inducing differentiation of PSCs^122^; Figure 4). NEUROG/NEUROD pioneering factors can bind heterochromatin and induce euchromatin, leading to chromatin activation and further neural differentiation, but their binding is DNA methylation-dependent as they can bind solely to unmethylated DNA.^123,124^ Thus, DNA hypermethylation in AT/RTs compromises their function, both directly and indirectly, via suppressed expression.^62,125^ The hypermethylated DNA regions identified on AT/RTs are likely to block neural differentiation, maintaining AT/RT cells in a progenitor-like state. Some of the DNA methylation patterns in AT/RTs were shown to be similar to those in pluripotent stem cells and harbored binding sites for neural transcription factors, further indicating halted cell differentiation and stem-like state (Figure 4).^62^ However, AT/RT-unique DNA hypermethylation was also observed, which was associated with EZH2 and neuronal SMARCA4 binding sites, as well as some of the downregulated genes such as *TCEA3*, *NEUROG1*, and *NEUROD2* (Figure 4).^62^

The distinctive characteristics of AT/RTs, such as DNA methylation signatures resembling pluripotency^62,126^ suggest potential therapeutic approaches using DNA methyltransferase (DNMT) inhibitors (Figure 4). The application of small-molecule inhibitors, such as decitabine (5-Aza-2’-deoxycytidine), which has already been shown to penetrate the blood-brain barrier^127^, has shown significant effects on AT/RT-MYC and AT/RT-SHH cell lines. Furthermore, in vivo studies have demonstrated reduced tumor growth in both primary tumors obtained from ecMRT and patient-derived relapsed ecMRT xenograft models.^70^ Additionally, in a recent study conducted on patients with AT/RT, approximately 30% showed signs of antitumor activity after decitabine treatment.^128^ While the study cohort size was moderate and lacked patients from different subgroups, the use of DNMT inhibitors might provide additional therapeutic strategies for patients with AT/RT.

These results suggest that blockage of neural differentiation driven by the dysregulation of epigenetic mechanisms is essential for AT/RT tumorigenesis. The NEUROD/NEUROG family of pioneer factors appears to play an important role in this process along with the Notch pathway. However, the exact mechanisms and key players regulating differentiation blockage remain partly unresolved. For instance, it remains unclear whether the mechanisms differ between AT/RT subgroups, as the apparent vulnerabilities to specific inhibitors would suggest.^51,58,59^ Further research is required to determine the exact mechanisms of action and optimal therapeutic approaches.

## Discussion

AT/RTs are challenging pediatric brain tumors with high mortality rates. While AT/RTs carry only a few recurrent alterations and are almost devoid of genetic heterogeneity, distinct tumor subgroups with epigenetic and transcriptional heterogeneity have been observed.

The antagonistic nature of mSWI/SNF and PRC2 complexes could offer an explanation for AT/RT development: with *SMARCB1* loss leading to a significant reduction in mSWI/SNF function, the PRC2 complex is left unregulated, thereby silencing crucial genes via repressive histone modifications. However, it has become clear that this is not the sole mechanism underlying AT/RT malignancy, evident from a global decrease in histone levels in these tumors. While some regions gain repressive histone methylation and exhibit PRC2-mediated downregulation, emerging evidence of DNA hypermethylation in relevant regulatory regions for neural differentiation cannot be overlooked.

Part of the AT/RT genome exhibits DNA hypermethylation that resembles the patterns found in pluripotent stem cells. The location of these stem-like DNA hypermethylation patterns in neural cell differentiation-related regulatory regions suggests an inability to remove DNA methylation, thus hindering the normal progression of lineage commitment and differentiation. Defects in displacing existing DNA methylation after *SMARCB1* loss would render the cellular context highly relevant for AT/RT development. The prevailing epigenetic regulation maintaining the cells in an undifferentiated state would make the cell-of-origin a contributor to AT/RT malignancy. In this scheme, subgroup-related differences would emerge, at least in part, from the cellular context.

However, there are also unique methylation patterns specific to AT/RT that cannot be explained just by the inability to remove DNA methylation. The re-expression of SMARCB1 or SMARCA4 has been shown to induce changes in gene expression similar to DNA methyltransferase inhibition, indicating cross-talk between mSWI/SNF and DNA methyltransferases.^70,129^ Nonetheless, further research is needed to conclusively determine how the loss-of-function of mSWI/SNF contributes to the placement of DNA methylation, and to the causative standstill in cell differentiation. While it seems likely that both histone and DNA methylation plays a role in differentiation blockage, it remains unclear to what degree these processes are independent or cooperative. In addition, ncBAF activity appears to be a relevant driver of oncogene activation at least in the context of ecMRTs, and similar findings are likely to be recapitulated also in AT/RT. Further research will help us better understand the development and regulation of AT/RTs and the neural cell differentiation process in general, which will facilitate uncovering novel, effective therapeutic strategies for AT/RTs.

## Data Availability

No datasets were generated or analyzed during the current study.
